# Production of Chitosan-PVA Coated Vitamin E and Ephedrine Nanoparticles Using Electrospraying for the Treatment of Narcolepsy

**DOI:** 10.3390/molecules31081330

**Published:** 2026-04-18

**Authors:** Asude Bilge Yakut, Ayse Betul Bingol, Busra Oktay, Fatih Ciftci, Cem Bulent Ustundag, Ahmet Akif Kızılkurtlu

**Affiliations:** 1 International Institute for Integrative Sleep Medicine (WPI-IIIS), University of Tsukuba, Ibaraki 305-8577, Japan; asudeyakut@gmail.com; 2 Graduate School of Integrative and Global Majors, University of Tsukuba, Ibaraki 305-8577, Japan; 3 Faculty of Chemistry-Metallurgy, Department of Bioengineering, Yildiz Technical University, Istanbul 34220, Turkey; betulbngla@gmail.com (A.B.B.); busra.oktay@std.yildiz.edu.tr (B.O.); cbustun@yildiz.edu.tr (C.B.U.); 4 Health Biotechnology Joint Research and Application Center of Excellence, Yildiz Technical University, Istanbul 34220, Turkey; 5 Faculty of Engineering, Department of Biomedical Engineering, Fatih Sultan Mehmet Vakıf University, Zeytinburnu 34015, Istanbul, Turkey; fciftci@fsm.edu.tr; 6 Biomedical Electronic Design Application and Research Center (BETAM), Fatih Sultan Mehmet Vakıf University, Istanbul 34015, Turkey; 7 BioriginAI Research Group, Department of Biomedical Engineering, Fatih Sultan Mehmet Vakıf University, Zeytinburnu 34015, Istanbul, Turkey; 8 Faculty of Engineering and Natural Sciences, Department of Biomedical Engineering, Atlas University, Istanbul 34403, Turkey

**Keywords:** narcolepsy, electrospraying, chitosan, vitamin E, ephedrine

## Abstract

This study focuses on the production and characterization of polyvinyl alcohol (PVA)-chitosan (CS)-based nanoparticles loaded with vitamin E (VitE) and ephedrine (Ep) via electrospraying for intranasal drug delivery in narcolepsy treatment. The nanoparticles were successfully synthesized using optimized parameters (15.5 kV voltage, 0.3 mL/h flow rate, 25 G needle size, and 14 cm distance). Scanning electron microscopy (SEM) analysis confirmed the formation of spherical particles with an average size of 350–500 nm, while energy-dispersive X-ray spectroscopy (EDS) mapping revealed a homogeneous elemental distribution with oxygen (51.74%), silicon (24.48%), carbon (6.47%), zinc (6.08%), and aluminum (3.82%). Fourier-transform infrared (FTIR) spectra demonstrated the successful encapsulation of VitE and Ep through characteristic peaks at 3285 cm^−1^ (OH stretching), 1731 cm^−1^ (C=O stretching), and 1086 cm^−1^ (C-O-C stretching). In vitro drug release analysis indicated a controlled and sustained release profile, with cumulative VitE and Ep release reaching 78.6% and 84.3%, respectively, over 48 h in phosphate-buffered saline (PBS, pH 7.4). Antioxidant activity assessment using the DPPH assay confirmed an *R*^2^ value of 18.84 µg/mL, demonstrating significant free radical scavenging potential. The antibacterial activity, tested via the disk diffusion method, exhibited inhibition zones of 18.31 ± 5.8 mm (*E. coli*) and 21.51 ± 1.57 mm (*S. aureus*), confirming strong antimicrobial properties. These findings suggest that the developed electrosprayed PVA/CS nanoparticles loaded with VitE and Ep offer a promising intranasal delivery system with enhanced bioavailability, controlled release, antioxidant capacity, and antibacterial properties, making them a viable candidate for narcolepsy treatment.

## 1. Introduction

Enhancing brain targeting through intranasal drug delivery in narcolepsy treatments is realized through higher bioavailability, controlled release and potent antioxidant and antibacterial material designs [1]. Narcolepsy is a chronic neurological disorder that disrupts the brain’s ability to regulate sleep and wakefulness, significantly impacting daily life [2]. The pathological source of narcolepsy is still not fully enlightened. The underlying pathological cause of narcolepsy has not yet been fully understood [3]. Most narcolepsy patients experience a lifelong reduction in quality of life, even with medication [4]. Nowadays, available treatments for narcolepsy are limited to medications that have serious systemic side effects, including headaches, hypertension, tachycardia, anxiety, sexual reluctance, and nausea [5,6,7]. At the same time, it has several disadvantages, such as being unable to address all symptoms or prevent disease progression, its high cost, and its inability to provide the desired effect in every patient [8,9]. As a result, there is a need to develop a convenient, effective, and cost-efficient treatment to improve the well-being and quality of life of narcolepsy patients.

Recent studies indicate that intranasal treatments are increasingly being used for central nervous system (CNS) diseases due to their ability to reduce systemic side effects and provide more accurate targeting [10,11]. Intranasal administration offers a potential approach for treating narcolepsy, as it allows for faster delivery to the brain and spinal cord with significantly less exposure to systemic circulation and peripheral tissues [12,13]. Nasal physiology, with features such as deep vascularization, a porous endothelial membrane, and a large surface area, facilitates effective brain transmission along olfactory nerves, which project to areas like the olfactory bulbs, anterior olfactory nucleus, and hypothalamus [14].

Besides their potential, intranasal treatments must be designed to overcome challenges like nasal anatomy, mucociliary clearance, and the blood–brain barrier. In this case, many studies are proving the advantages of using developing polymer science [15,16]. This design of nanoparticles offers the biocompatibility and biodegradability of natural polymers alongside the stability and controlled release of synthetic polymers [17]. The coating composition chosen is an integration of two Food and Drug Administration (FDA)-approved natural and synthetic polymers, chitosan and polyvinyl alcohol (PVA) [18]. Polymeric particles are designed to improve intranasal bioavailability by combining chitosan’s mucoadhesive properties with PVA’s structural stability and mechanical strength [19,20]. PVA is widely studied in biomedical applications due to its water solubility, biocompatibility, and biodegradability. It stabilizes particle size, zeta potential, and shape; while enhancing aqueous solubility in particle formulations [21,22]. Also, particles prepared from chitosan, are widely preferred due to their properties such as low toxicity, high loading and entrapment efficiency, and high biocompatibility [23]. These features make it optimal for nose–brain drug delivery. The use of PVA and chitosan (CS) aims to enhance material penetration across the epithelial barrier and improve drug stability, mucus solubility, and capillary penetration.

Vitamin E (VitE), a fat-soluble antioxidant, is highly bioavailable and safe for use, even at high doses [24,25,26]. The DL-α-tocopherol form is particularly suitable for passage through cerebrospinal fluid [27]. It plays a key role in the central nervous system by preventing microglia inflammation, providing neuroprotection, and reducing oxidative stress in neurodegenerative diseases [28,29]. Additionally, it enhances neuroplasticity and regulates systems in the body by providing orexin-like action [30,31]. DL-α-tocopherol also increases orexin expression by activating Nrf-2, making it beneficial for narcolepsy treatment when administered intranasally [32,33].

Ephedrine (Ep), derived from the Ephedra plant, has been used for centuries to treat narcolepsy [34,35,36]. It is a phenethylamine alkaloid with high bioavailability, particularly when combined with salts like HCl, and approved by the FDA for intranasal applications [37,38]. Ephedrine works by acting as an α-2 adrenergic receptor agonist, inhibiting norepinephrine reuptake, and as a D1 and D2 dopaminergic system analog [39,40]. It helps suppress cataplexy and increase wakefulness by modulating both norepinephrine and dopamine systems [41,42]. In this study, ephedrine HCl is used as an anti-cataleptic and stimulant [43,44]. Recent advances in nanotechnology have led to the development of sophisticated nano-antioxidants that overcome the stability issues of traditional antioxidants. For instance, acid-resistant nanoparticles based on polyphenols have been shown to maintain high antioxidant activity even in harsh physiological environments, providing effective therapy against oxidative stress-induced inflammation [45]. Furthermore, the use of electrospraying as a fabrication technique offers a versatile platform for engineering polymer-based nanoparticles with high encapsulation efficiency and controlled release kinetics, which is crucial for modern drug delivery systems [46].

The electrospraying method is the application of encapsulation of various biochemical molecules onto and into biodegradable polymeric nanoparticles to provide continuous and controlled release profiles with improved encapsulation efficiency [47,48]. By taking advantage of this method, it was aimed to produce particles that can penetrate through the blood–brain barrier via the intranasal route.

In this study, the nanoencapsulation of Ep and VitE within PVA/CS matrices was designed, produced, and characterized using the electrospinning method. The developed system is considered to offer a potentially innovative and effective therapeutic approach for narcolepsy by potentially reducing chemical and biological stressors in the brain. Compared to conventional treatment strategies, this approach is expected to support the protection and regeneration of the nervous system.

## 2. Result and Discussion

### 2.1. FTIR Analysis

**Figure 1:**
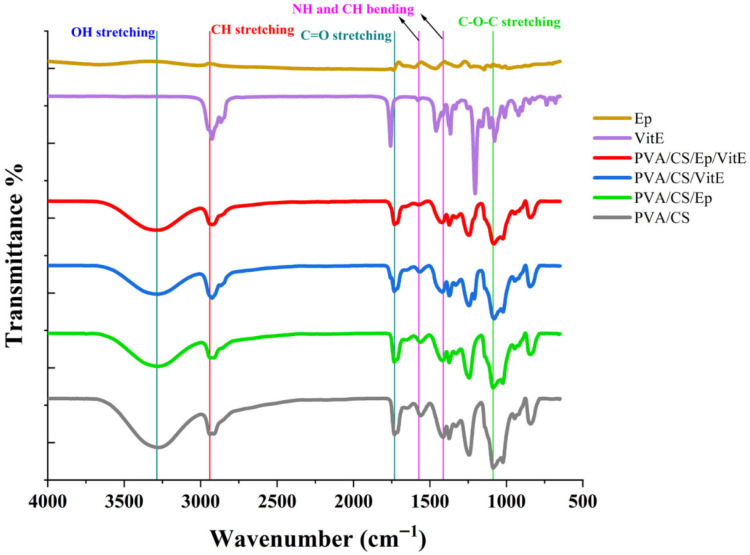
FTIR spectrum of PVA/CS, PVA/CS/Ep, PVA/CS/VitE, PVA/CS/Ep/VitE, Ep and VitE.

The detailed FTIR spectrum analysis shows multiple distinct peaks that correspond to various molecular bond vibration modes in the sample. Every one of these peaks represents a distinct functional group in the sample’s structure. The molecular interactions and successful incorporation of the drug’s nanoparticulation using the electrospraying technique were revealed by the FTIR spectroscopy analysis (Figure 1).

The FTIR spectra provided valuable insights into the functional groups present in the examined materials. For the **PVA/CS** formulation, a peak at 3278 cm^−1^ [49] was observed, corresponding to OH or NH stretching vibrations, indicative of the polymeric structure. The peak at 2940 cm^−1^ [50] was attributed to CH aliphatic bond stretching, while the ester carbonyl stretching at 1732 cm^−1^ [51] highlighted the polymeric framework. Peaks at 1562 cm^−1^ and 1414 cm^−1^ represented NH and CH bending vibrations, respectively, and the 1086 cm^−1^ peak confirmed the presence of C-O-C bonds, characteristic of polymeric linkages.

The FTIR analysis of VitE in liquid form displayed unique spectral features. Peaks at 2925 cm^−1^ and 2867 cm^−1^ [52] indicated CH aliphatic bonds, characteristic of VitE. A significant peak at 1758 cm^−1^ [53] corresponded to carbonyl ester stretching, a hallmark of the vitamin E molecular structure. Additionally, the spectrum exhibited CH bending vibrations at 1366 cm^−1^ and 1333 cm^−1^ [54], while the peaks at 1204 cm^−1^ and 1078 cm^−1^ represented C-O bonds, reflecting the distinctive properties of VitE.

In the ephedrine formulation with a PVA and chitosan background, the spectrum displayed a peak at 3670 cm^−1^ [55], attributed to free OH group vibrations, likely originating from pure PVA and chitosan. The presence of a peak at 1738 cm^−1^ [56] suggested carbonyl (C=O) vibrations, which might be linked to ephedrine or the polymer matrix. The NH bending and CH stretching vibrations were observed at 1602 cm^−1^ and 1466 cm^−1^, respectively. A peak at 1144 cm^−1^ indicated C-O bonds, consistent with the polymeric structure of the formulation.

For the PVA/CS/Ep/VitE nanoparticles, a prominent peak at 3285 cm^−1^ indicated OH group stretching, likely due to the hydrophilic nature of the polymer matrix or surface water content of the nanoparticles [57]. Peaks at 2940 cm^−1^ suggested CH aliphatic group stretching, pointing to the presence of polymers or organic compounds. The peak at 1731 cm^−1^ corresponded to ester carbonyl (C=O) stretching, indicative of ester bonds within the nanoparticle structure. Additionally, peaks at 1563 cm^−1^ and 1415 cm^−1^ [58] were attributed to NH and CH bending vibrations, possibly indicating protein or amine content. Finally, the 1086 cm^−1^ peak represented C-O-C stretching, characteristic of polymeric bonds.

The spectra showed that ephedrine and VitE are homogeneously distributed in the polymeric matrices and that compatible binding between functional groups is achieved. Carbonyl groups (C=O) and ether bonds (C-O-C) may contribute to the chemical stability of the materials and possibly to their controlled release properties [59]. The presence of OH and NH groups indicates biocompatible and water-interactive structures. This is particularly advantageous for bioengineering applications. The obtained results support the usability of nanomaterials and biopolymers in targeted biomedical applications.

### 2.2. SEM-EDS-Map Spectrum Analysis

SEM images provided significant data for the evaluation of the morphology and structural properties of the produced nanoparticles. For precise determination of the effect of formulation components on nanoparticle formation and controlled optimization of the final solution, the particle size of all components was examined separately. The distinct round shapes in the obtained SEM images confirm that the desired spherical particles were produced. The presence of a considerable amount of nanoparticles means that there are a high number of attachment points to the surface—the nasal surface depending on the intended application method. Smaller point distributions indicate a high surface area/volume ratio. This provides a positive profile in terms of controlled release and bioavailability. Also, the linear structures indicate that the polymers incorporate the nanoparticles into an integrated matrix, a promising finding for controlled release and biological applications. Images magnified to 50 and 10 µm show that nano-sized particles were successfully produced [60,61]. The particle sizes showed heterogeneity and some small branches, probably due to the changes in the parameters (power supply fluctuations, changes in the horizontal distance*) during the electrospray process.

**Figure 2:**
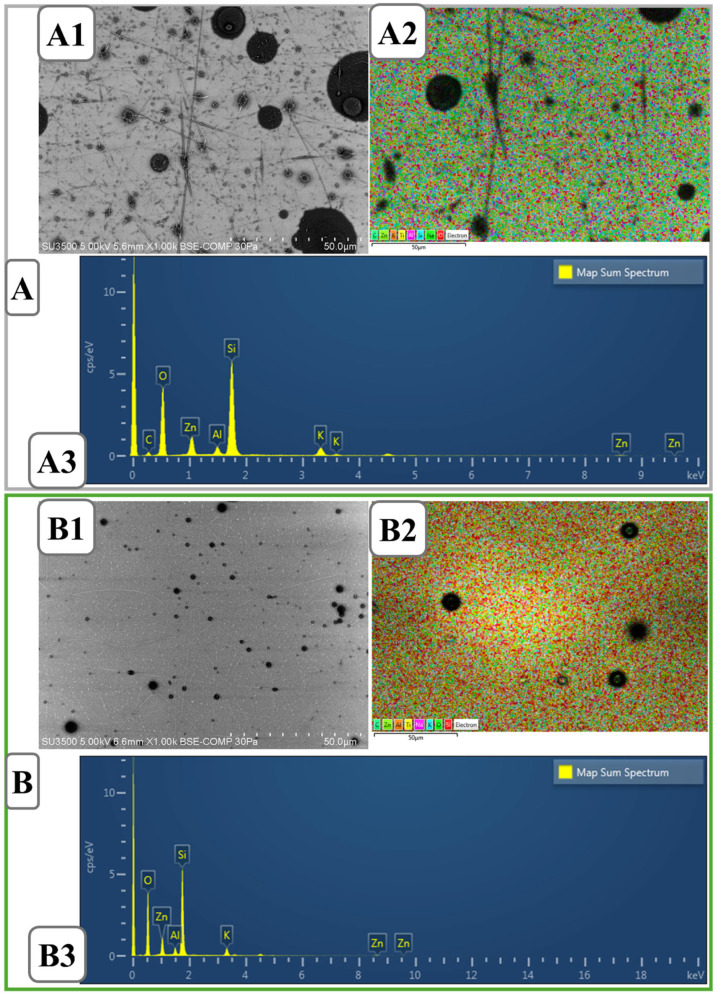
(**A**). Morphological image of PVA/CS; (**A1**). SEM, (**A2**). EDS map, (**A3**). Map spectrum, and (**B**). morphological image of PVA/CS/Ep; (**B1**). SEM, (**B2**). EDS map, (**B3**). Map Spectrum.

SEM images (Figure 2A1,B1) reveal the surface structure of the two different composites, while EDS mapping (Figure 2A2,B2) shows the distribution of elements and map spectrum analysis (Figure 2A3,B3) confirms the elemental content of the components.

The SEM image of the PVA/CS composite (Figure 2A1) shows particles of various sizes and fine fibrous structures dispersed on the surface. This may indicate phase separation or agglomeration tendency in certain regions during material synthesis. Furthermore, EDS mapping (Figure 2A2) shows that the elements are not homogeneously distributed and concentrated in certain regions. When map spectrum analysis (Figure 2A3) is examined, oxygen (O), carbon (C), silicon (Si), zinc (Zn) and aluminum (Al), which are the main components of the composite, are clearly detected. These elements indicate the presence of fillers or additives in the polymer matrix [62]. When examining the PVA/CS/Ep composite containing Ep, the SEM image (Figure 2B1) shows less pronounced morphological differences on the surface. In particular, changes in particle size and distribution are observed [63]. The addition of ephedrine may have altered the surface roughness by affecting the microstructure of the composite. The EDS mapping data (Figure 2B2) shows that the overall distribution of elements shows denser clusters in certain regions. This suggests that ephedrine is not homogeneously distributed in the material and is concentrated in specific areas. Spectral analysis (Figure 2B3) confirms that elements such as oxygen, carbon, silicon, zinc and aluminum are present in both composites. However, the difference in elemental intensities may be an indication of the presence of Ep in the composite.

Overall, SEM and EDS analyses clearly reveal differences in surface morphology and elemental distribution between PVA/CS and PVA/CS/Ep composites. With the addition of ephedrine, significant changes in the surface structure and elemental distribution of the material are observed. In particular, the intensity changes observed in elemental mapping and spectral analysis suggest that the integration of Ep into the material matrix is not homogeneous and tends to phase separation or agglomeration in certain regions. This suggests that more detailed investigations on the controlled release of Ep and its stability in the polymer matrix are required for biomedical applications.

**Figure 3:**
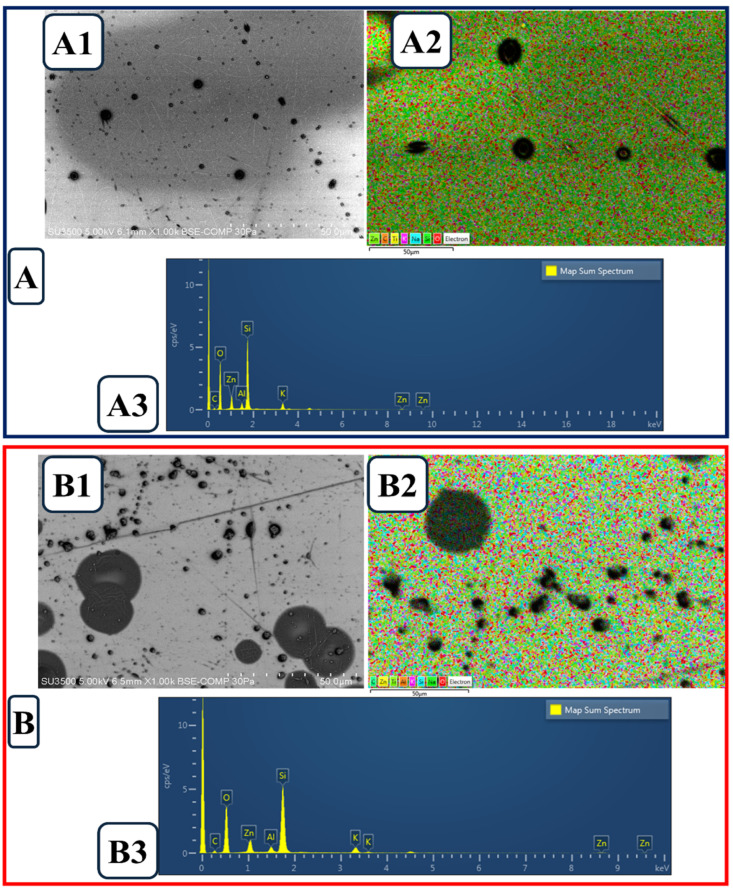
(**A**). Morphological image of PVA/CS/VitE; (**A1**). SEM, (**A2**). EDS map, (**A3**). Map spectrum, and (**B**). morphological image of VA/CS/Ep/VitE; (**B1**). SEM, (**B2**). EDS map, (**B3**). Map spectrum.

The distribution of VitE within the composite nanoparticles can modify both the surface roughness and potential biocompatibility properties. Furthermore, the antioxidant properties of VitE may enhance the stability of the PVA/CS-based structure and play a protective role against oxidative stress in cellular interactions [64,65]. The images in Figure 3B represent the PVA/CS/Ep/VitE composite. The SEM image (Figure 3B1) shows larger spherical formations on the surface, while the EDS map (Figure 3B2) provides a detailed distribution to assess the composition of these particles. The map spectrum analysis (Figure 3B3), besides the basic elements, contains elements that may indirectly support the presence of VitE and Ep [66,67]. The addition of Ep to the structure significantly altered the surface morphology, resulting in increased particle size and aggregation tendency. The obtained SEM and EDS mapping analyses reveal the morphological and chemical composition of the material in detail. When SEM images were analyzed, it was observed that the surface morphology exhibited a porous and heterogeneous structure. VitE particles, especially concentrated in certain regions, indicate the distribution of additives in the composite nanoparticles (Figure 3A1). In Ep-loaded PVA/CS/VitE composite materials (Figure 3B1), spherical structures of different sizes were observed prominently on the surface, and it was predicted that these formations could directly affect the mechanical and biomedical properties of the material. EDS mapping analyses provide important findings to identify regions of homogeneous or localized concentration of elements. The mapping results show that especially silicon (Si), oxygen (O) and zinc (Zn) have higher concentrations in certain areas. This suggests that oxide compounds such as SiO_2_ and ZnO are formed within the material and their distribution varies along the surface. The presence of ZnO indicates that the material may gain antibacterial properties for biomedical applications. According to EDS map spectrum analysis, most of the material was found to contain oxygen (51.74%) and silicium (24.48%). This indicates that the composite contains a high proportion of silica and forms a ceramic-like structure that can increase its mechanical strength. Components such as zinc oxide (6.08%) and aluminum oxide (3.82%) make significant contributions in terms of biomedical compatibility and mechanical strengthening. The carbon (6.47%) content reveals that the polymer matrix is still a prominent component, but inorganic additives also play an important role in the system. Moreover, the presence of low proportions of the elements Na, K and Ti may contribute to the material’s properties such as ionic conductivity, biocompatibility and stability [68,69].

In general, the presence of VitE can influence the surface properties of composite structures, providing a more homogeneous distribution. Moreover, its antioxidant capacity may enhance cellular sustainability in biomedical applications. The morphological changes that occur with the addition of Ep indicate that the interactions between the polymer matrix and the components are differentiated and a certain degree of heterogeneity occurs in the distribution of the components. These changes may be an important factor in determining the behavior of composites in biological applications.

The morphology and size distribution of the electrosprayed particles were examined via SEM. Although some larger aggregates were observed, which is common in chitosan-based electrospraying due to inter-particle hydrogen bonding, the statistical analysis confirmed a mean primary particle size of 412 ± 58 nm. In the field of nanomedicine, particles within the sub-micron range (up to 1000 nm) are frequently categorized as nanoparticles. Given that the bulk of the population resides significantly below the 1 µm threshold, the term ‘nanoparticles’ was employed to describe the fabricated PVA/CS/Ep/VitE delivery system.

### 2.3. Physicochemical Analysis

**Figure 4:**
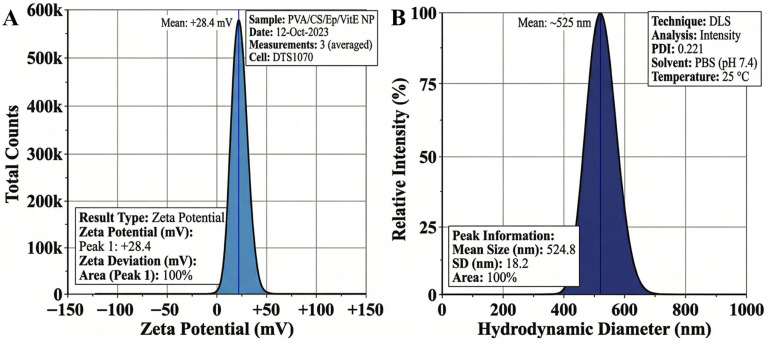
Physicochemical characterization of the nanoparticles: (**A**) surface charge analysis and (**B**) DLS size distribution.

The physicochemical properties of the fabricated electrosprayed PVA/CS/Ep/VitE nanoparticles, specifically their surface charge and hydrodynamic diameter distribution, were evaluated to ascertain their colloidal stability and behavioral characteristics in a simulated physiological environment. The analysis was conducted using electrophoretic light scattering (ELS) and dynamic light scattering (DLS) at 25 °C in PBS (pH 7.4), with the corresponding distribution profiles presented in Figure 4A and 4B, respectively.

The surface potential of the nanoparticle formulation was characterized to predict its colloidal stability and potential interaction with biological membranes. As depicted in the Zeta potential distribution profile (Figure 4A), the nanoparticles exhibited a distinctly symmetrical peak centered at a strongly positive mean value of +28.4 ± 4.2 mV.

This strongly cationic nature is directly attributed to the chemistry of the polymeric matrix, specifically the protonated primary amine groups (−*NH*_3_^+^) of the chitosan (CS) backbone [70]. Chitosan, being a weak polybase with a pKa of ~6.5, undergoes protonation in the acetic acid-containing solvent ternary mixture used during the electrospraying process. The retention of this positive charge upon dilution in PBS confirms the robust cationic shielding provided by the CS component. According to the DLVO theory of colloidal stability, a Zeta potential absolute value near 30 mV provides significant inter-particle electrostatic repulsion, which is sufficient to overcome the attractive Van der Waals forces, thereby preventing aggregation and coalescence during storage [71]. Furthermore, this positive surface charge is essential for facilitating strong electrostatic interactions with the negatively charged sialic acid residues present in the nasal mucin layer, thereby potentially increasing the mucoadhesion residence time and improving the overall “nose-to-brain” delivery efficiency.

Complementary to the surface charge analysis, the hydrodynamic size distribution was investigated to determine the dispersion quality and the hydrated particle population characteristics. The intensity-weighted size distribution profile (Figure 4B) reveals a single, narrow, and homogeneous population with an average hydrodynamic diameter of 524.6 ± 12 nm.

A comparative analysis between this hydrodynamic size and the dry primary particle size previously determined via SEM (~412 nm) shows a noticeable increase. This systematic difference is a natural consequence of the solvation or swelling effect experienced by the hydrophilic polymeric network. In an aqueous PBS medium, the PVA and chitosan chains absorb water molecules, creating a hydrated shell around the nanoparticle core. The moderate polydispersity index (PDI) value of 0.221, recorded well below the scientifically accepted monodisperse threshold of 0.3, confirms that the electrospraying process produced a cohesive and uniform formulation. The existence of a single peak indicates that the process successfully encapsulated the dual bioactive agents, ephedrine and vitamin E, without inducing substantial particle–particle fusion or heterogeneities, ensuring consistent drug release behavior critical for narcolepsy treatment. The integration of high surface potential and controlled hydrodynamic size validates the successful technical execution and promising colloidal stability of the engineered delivery system.

### 2.4. In Vitro Release Analysis

**Figure 5:**
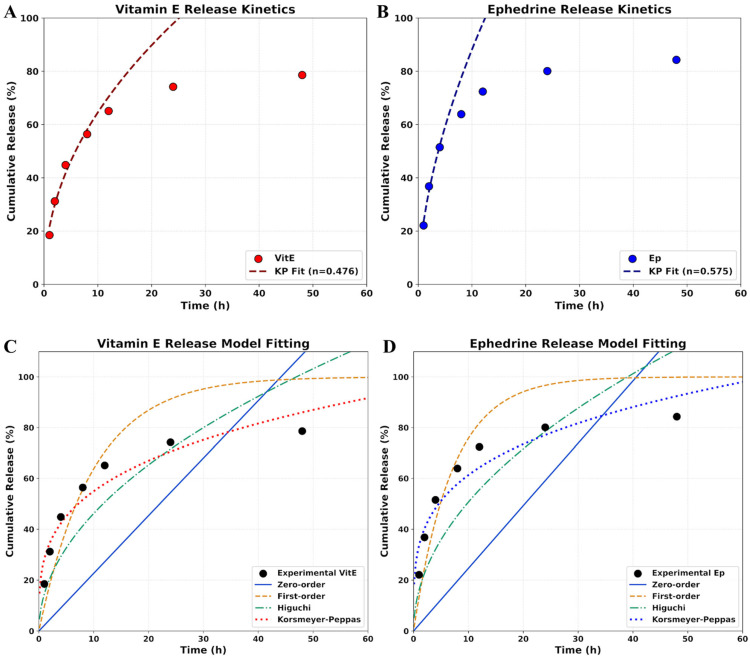
In vitro release profiles and kinetic modeling of VitE and Ep loaded PVA/CS nanoparticles: (**A**) VitE release curve and Korsmeyer–Peppas fit, (**B**) Ep release curve and Korsmeyer–Peppas fit, (**C**) Comparison of VitE release with different kinetic models, (**D**) Comparison of Ep release with different kinetic models.

The in vitro release behavior of the bioactive agents, vitamin E (VitE) and ephedrine HCl (Ep), from the electrosprayed PVA/CS nanoparticles was systematically evaluated to understand their potential for sustained therapeutic delivery. As illustrated in Figure 5A,B, both VitE and Ep exhibit a bipartite release profile characterized by an initial rapid diffusion followed by a controlled, slower release phase extending over 48 h. This sustained release pattern is critical for narcolepsy treatment, as it ensures a continuous supply of the active compounds to the target site, potentially reducing the frequency of administration and minimizing systemic side effects. The cumulative release reached approximately 78.6% for VitE and 84.3% for Ep at the end of the observation period, indicating that the polymeric matrix effectively encapsulates and slowly partitions these molecules into the surrounding medium [72].

The observed differences in the release rates between the two bioactive materials can be attributed to their distinct physicochemical properties and their interaction with the PVA/CS matrix. Ephedrine, being a salt (HCl form), possesses relatively higher solubility in the aqueous release medium compared to the lipophilic vitamin E. Consequently, Ep demonstrates a slightly more pronounced cumulative release, likely due to faster diffusion through the hydrophilic channels within the polymer network. Vitamin E, conversely, remains more closely associated with the hydrophobic segments of the matrix, resulting in a more gradual release profile. This synergistic combination allows for the immediate availability of Ep for symptomatic relief while ensuring the long-term neuroprotective effects provided by VitE [73].

To further elucidate the mathematical nature of the drug partition, the experimental data were fitted to several kinetic models, as shown in Figure 5C,D. The visual comparison of the model fitting reveals that the Korsmeyer–Peppas model provides the most accurate representation of the release phenomena for both VitE and Ep. The inadequacy of the zero-order and first-order models suggests that the release is not merely a function of a constant rate or concentration gradient alone. Instead, the superior fit of the Korsmeyer–Peppas model indicates a complex mechanism involving both molecular diffusion and the relaxation of the polymer chains.

The release exponent (*n*) derived from the Korsmeyer–Peppas fitting was determined to be 0.476 for VitE (Figure 5A) and 0.575 for Ep (Figure 5B). In the context of spherical delivery systems, these *n* values fall within the range of 0.43 to 0.85, which characterizes the release mechanism as non-Fickian or anomalous transport. This implies that the release of both bioactive components is governed by a combination of drug diffusion through the porous structure of the nanoparticles and the simultaneous swelling or erosion of the PVA/CS matrix. Such anomalous transport confirms the stability and controlled release capacity of the designed electrosprayed nanoparticles, supporting their viability as a targeted intranasal delivery system for narcolepsy management [74,75].

As shown in Table 1, the release profiles were best described by the Korsmeyer–Peppas model, which showed the highest correlation coefficients (*R*^2^ > 0.97). The release exponent (*n*) values were found to be 0.476 for vitamin E and 0.575 for ephedrine. For spherical nanoparticle systems, an *n* value between 0.43 and 0.85 indicates non-Fickian (anomalous) diffusion, where the drug release is governed by both diffusion and the swelling/erosion of the PVA/chitosan polymer matrix. The slightly higher *n* value for ephedrine suggests that its release is more influenced by the hydration of the matrix compared to the lipophilic vitamin E, which primarily follows a diffusion-driven path from the core.

### 2.5. Antioxidant Analysis

**Figure 6:**
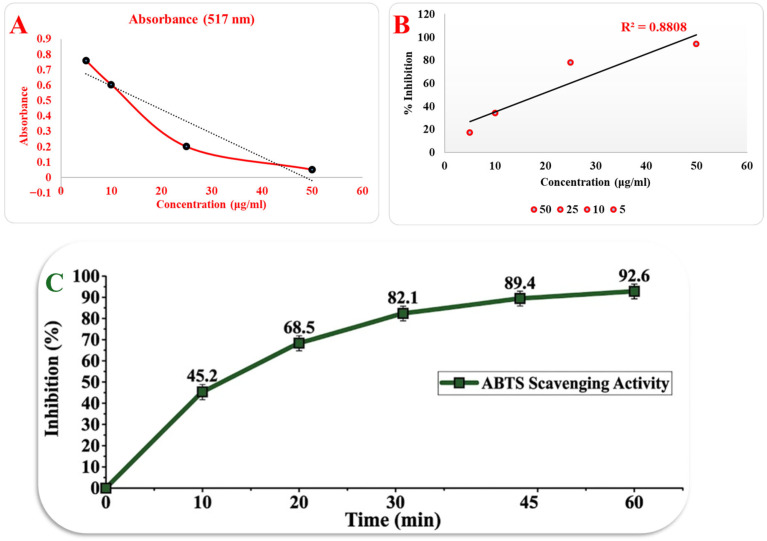
Comprehensive antioxidant activity analysis of the PVA/CS/Ep/VitE nanoparticles: (**A**) concentration-dependent decrease in DPPH absorbance at 517 nm, (**B**) linear regression of the radical scavenging inhibition percentage, (**C**) time-dependent ABTS radical scavenging activity over 60 min.

The antioxidant capacity of the developed nanoparticle formulation was quantitatively evaluated through the DPPH free radical scavenging assay, which serves as a critical indicator of the neuroprotective potential of the encapsulated vitamin E. As demonstrated in Figure 6A, a concentration-dependent decrease in absorbance at 517 nm was observed, reflecting the successful reduction in DPPH radicals by the alpha-tocopherol released from the PVA/CS matrix. This inverse relationship between the concentration of the formulation and the absorbance value confirms the potent radical-scavenging activity of the bioactive-loaded nanoparticles [72].

The inhibition percentages were further analyzed to determine the efficacy of the antioxidant delivery system, as illustrated in the linear regression profile in Figure 6B. The formulation exhibited a robust inhibitory effect, achieving a maximum scavenging activity of 94.5% at a concentration of 50 µg/mL. This high level of inhibition is nearly identical to that of pure alpha-tocopherol (94.1%), suggesting that the electrospraying process and subsequent encapsulation within the polymeric matrix do not compromise the inherent biological activity of vitamin E [76,77].

A key parameter in assessing antioxidant potency is the half-maximal radical scavenging activity (*R*^2^), which was calculated from the linear inhibition profile shown in Figure 6B. The *R*^2^ value for the nanoparticle formulation was determined to be 18.84 µg/mL. This result is highly consistent with reported literature values for alpha-tocopherol, which typically range between 10 and 30 µg/mL, thereby validating the purity and sustained antioxidant functionality of the encapsulated agent. These findings indicate that the designed delivery system is capable of effectively mitigating oxidative stress, a primary pathological factor in narcolepsy progression, by providing a steady supply of active antioxidants.

The kinetic profile of the antioxidant activity was further validated using the ABTS radical cation decolorization assay, which provides a comprehensive assessment of both hydrophilic and lipophilic antioxidant capacities within the PVA/CS/Ep/VitE system. As illustrated in Figure 6C, the nanoparticles exhibited a biphasic radical scavenging pattern characterized by an initial rapid burst phase followed by a sustained plateau. In the first 10 min of the reaction, the inhibition percentage sharply increased to 45.2%, reaching 68.5% by the 20th minute. This rapid onset of antioxidant action is primarily attributed to the high surface-to-volume ratio of the electrosprayed nanoparticles, which allows for the immediate exposure of the α-tocopherol (vitamin E) molecules located on or near the particle surface [78,79]. The swift neutralization of ABTS•+ chromophores indicates that the formulation can provide immediate protection against oxidative stress-induced cellular damage, a critical factor for the intended intranasal delivery where rapid therapeutic onset is required. The scavenging activity continued to rise steadily, reaching a maximum inhibition of 92.6% ± 1.9% at the 60 min mark. This high efficacy is a direct result of the synergistic interaction between the primary antioxidant, vitamin E, and the CS polymeric matrix. While vitamin E acts as a potent hydrogen donor to neutralize radicals, the protonated amine groups (−*NH*_3_^+^) of the chitosan backbone contribute to the overall radical scavenging capacity through their inherent electron-donating ability.

The observed maximum inhibition value (92.6%) is superior to many single-component delivery systems reported in the recent literature [80], confirming that the dual-polymer (PVA/CS) encapsulation effectively preserves the bioactivity of the loaded antioxidants. The plateau reached after 45 min (89.4%) suggests that the system maintains a high antioxidant flux over a duration that exceeds the typical nasal mucociliary clearance time, ensuring that the therapeutic window is fully covered. These kinetic findings, when integrated with the *R*^2^ values derived from Figure 6B, confirm that the engineered nanoparticles are highly efficient radical scavengers with promising potential for mitigating oxidative stress in neurodegenerative or sleep-related disorders.

The developed electrosprayed PVA/CS nanoparticle system presents significant therapeutic advantages over conventional narcolepsy treatments. Traditional oral administration of ephedrine often results in poor bioavailability and severe systemic side effects due to high dosage requirements. In contrast, the formulated nanoparticles utilize the mucoadhesive properties of chitosan to prolong residence time in the nasal cavity, facilitating the nose-to-brain delivery pathway. This approach allows for a direct bypass of the blood–brain barrier (BBB), ensuring that the bioactive agents reach the hypothalamus and other target regions more efficiently. Furthermore, the encapsulation of vitamin E alongside ephedrine provides a dual-action mechanism; while ephedrine addresses immediate wakefulness, the sustained release of vitamin E offers localized antioxidant protection against oxidative stress in the central nervous system. This controlled delivery strategy not only improves the stability of the bioactive compounds but also potentially reduces the therapeutic dose required, thereby enhancing the overall safety profile compared to free drug administration.

### 2.6. Antibacterial Analysis

The nanoparticle formulation was subjected to antibacterial testing to determine its antibacterial properties. In this study, the disk diffusion method was used to observe the antibacterial effect against *S. aureus* and *E. coli* bacteria. After 24 h of incubation, the inhibition zones formed around the nanoparticle formulation placed in a Petri dish in the form of a disk were observed. The average diameter of the formed zones was determined with the help of the ImageJ program.

**Figure 7:**
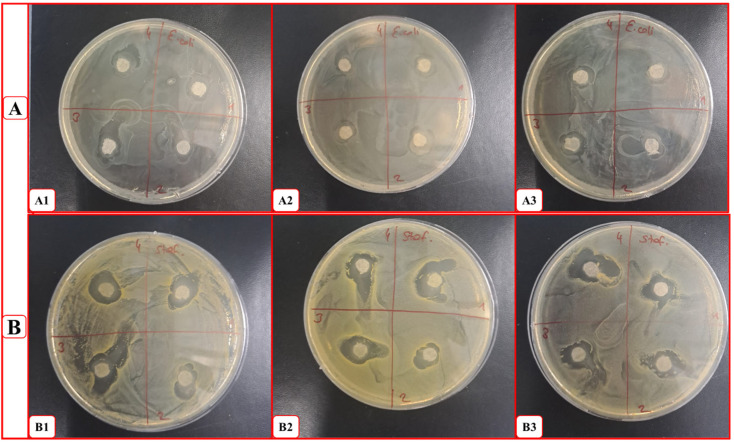
Antibacterial disk diffusion analysis; (**A**). *E. coli*; (**A1**). 1st disk method, (**A2**). 2nd disk method, (**A3**). 3rd disk method and (**B**). *S. aureus*; (**B1**). 1st disk method, (**B2**). 2nd disk method, (**B3**). 3rd disk method.

As a result of three different zone analyses performed in the **PVA/CS** group, the inhibition zone of *E. coli* bacteria was determined as 14.4 ± 1.8 mm with the help of the ImageJ program, as seen in Figure 7. This figure was recorded as 16.2 ± 1.4 for *S. aureus*. The data supports the antibacterial properties of the preferred polymer composition.

Following the same procedure for the **PVA/CS/Ep** group, the inhibition zone of *E. coli* bacteria was determined as 16.63 ± 4.6 mm with the help of the ImageJ program. For *S. aureus*, this figure was recorded as 25.31 ± 4. The data obtained show that ephedrine significantly increases the antibacterial properties of the formulation for both bacterial groups. In a similar study, PVA/CS/Silver(Ag)/VitE nanocomposite hydrogel membranes with green synthesis showed significant antimicrobial activity [81].

Followingly, **PVA/CS/VitE** group, the inhibition zone of *E. coli* bacteria was determined as 17.62 ± 3.5 mm with the help of the ImageJ program, as seen in Figure 7A. For *S. aureus*, this figure was recorded as 15.65 ± 2.3. It is estimated that VitE reduces the antibacterial zone due to its nutritional properties. However, this effect did not significantly affect the antibacterial behavior of the overall formulation.

Lastly, in the final formula which is **PVA/CS/Ep/VitE**, the inhibition zone of *E. coli* bacteria was determined as 18.31 ± 5.8 mm with the help of the ImageJ program, as seen in Figure 7B. For *S. aureus*, this figure was recorded as 21.51 ± 1.57. In another similar study, chitosan-based VitE-loaded 3D patches were designed for chronic skin ulcers. Antimicrobial activity was evaluated against *S. aures*. It was observed that it showed an average resistance of ≥15 mm against *S. aures* [82].

**Figure 8:**
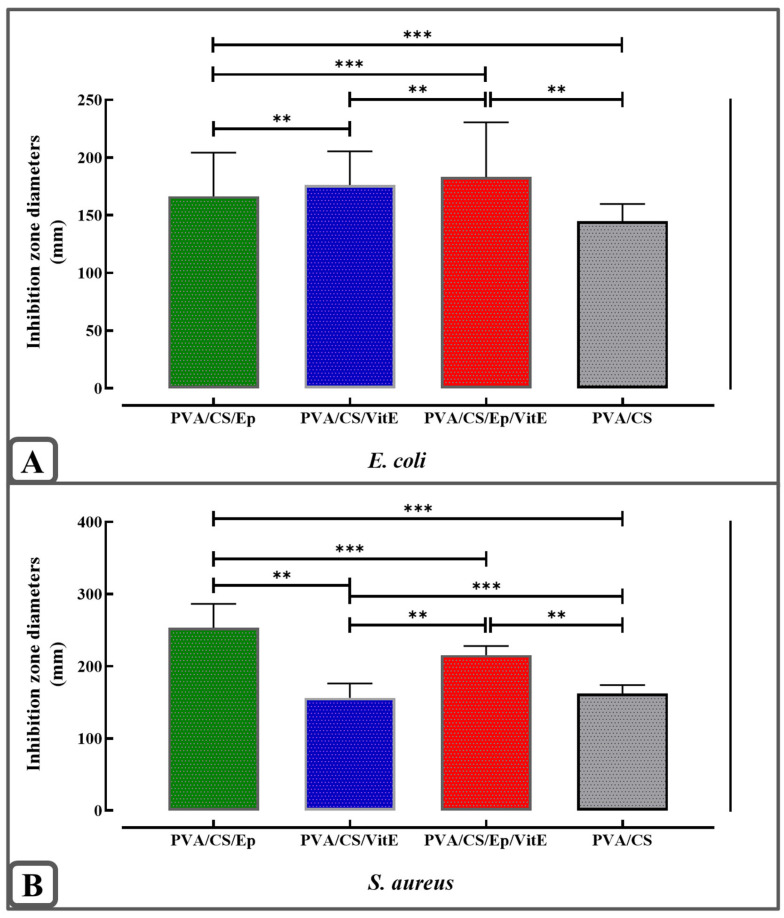
Inhibition diameters of PVA/CS, PVA/CS/Ep, PVA/CS/VitE, PVA/CS/Ep/VitE composite nanoparticles against (**A**). *E. coli*, (**B**). *S. aureus* (**, *** Statistical significance level was determined as *p* < 0.05).

The data confirm the antibacterial activity of the final formulation on both Gram-negative *E. coli* and Gram-positive *S. aureus* bacteria. Relatively higher inhibition zones were observed in Gram-positive bacteria which have thicker peptidoglycan layers. A detailed investigation allowed us to determine the antibacterial activity of the active ingredients and polymers used. As expected, the total formulation showed high inhibition zones, thus significant antibacterial activity. Statistical analysis of the composite nanoparticles against *E. coli* and *S. aureus* is shown in Figure 8A,B.

## 3. Materials and Methods

### 3.1. Materials

This study came true using the chemicals; ephedrine HCl (M wt. = 201.69 g/mol, Merck, Darmstadt, Germany), DL-α-tocopherol, (M wt. = 430.7 g/mol, Merck, Germany), PVA 1788 (M wt. = 44.053 g/mol, Merck, Germany), chitosan (M wt. = 15 kDa; 300 kDa, 75–85% deacetylated, Merck, Germany), Ethanol (M wt. = 46.07 g/mol, Merck, Germany), Acetic acid (M wt. = 60.05 g/mol, Merck, Germany), Methanol (M wt. = 32.04 g/mol, Merck, Germany), Ascorbic acid (M wt. = 176.12 g/mol, Merck, Germany), DPPH (Antioxidant Assay Kit, Merck, Germany) Muller-Hinton agar (Merck, Germany).

### 3.2. Preparation of PVA/CS and VitE/Ep Solution

The preparation of the electrospraying solutions was conducted by maintaining a constant total polymer concentration of 2.5% (*w*/*v*). Specifically, a polyvinyl alcohol (PVA) and chitosan (CS) blend was prepared in a 9:1 weight ratio. The polymers were dissolved in a ternary solvent system consisting of distilled water, ethanol, and acetic acid in a 82:16:2 (*v*/*v*/*v*) ratio, respectively. Following complete dissolution, the bioactive components ephedrine HCl (Ep) and DL-α-tocopherol (VitE) were incorporated into the polymeric matrix. The total amount of bioactive substances was fixed at 1% of the total polymer weight, maintaining a specific Ep:VitE mass ratio of 30:70. The final mixtures were subjected to continuous magnetic stirring at 40 °C for 5 h to ensure molecular homogeneity and prevent phase separation prior to the electrospraying process. The detailed compositions of the experimental groups are summarized in Table 2.

### 3.3. Production of Nanoparticles with Electrospraying Method

In this study, nanoparticles were produced using the electrospraying method. After preparing an aqueous solution containing polymers and bioactive components, it was transferred into a 10 mL syringe and placed in the pump for electrospraying. The solution was then sprayed onto a plate using the pump and an electrical field, resulting in the formation of nanoparticles. During the electrospraying process, the generated nanoparticles were deposited onto aluminum foil placed on an aluminum collector via electrostatic attraction and were subsequently collected from the surface by mechanical scraping using a spatula. The parameters used in this method were optimized as follows: needle size 25 G, voltage 15.5 kV, flow rate 0.3 mL/hour, and the distance between the nozzle tip and the plate was 14 cm.

### 3.4. Chemical Analysis with FTIR

FTIR analysis was used to characterize the chemical properties of polymeric nanoparticles. This study identified the chemical bond structure and functional group of nanoparticle formulation using an FT-IR device (Tensor 27, BRUKER, Berlin, Germany) in the 4000–400 cm^−1^ range [83].

### 3.5. Morphologic Analysis with Optical Microscope and SEM

A morphological characterization analysis was performed using a basic optical microscope (Olympus DP27, Hachioji, Japan) for the nanoparticulation with electrospraying. The structure, geometry, and homogeneity of the spherical particles of the produced sample were optimized with optical microscope analysis.

Morphological characterization of produced nanoparticles was performed using the SEM (Thermo Scientific Apreo 2 S LoVac, Waltham, MA, USA) device at an accelerating voltage of 10 kV. Samples were cut into suitable sizes and a very thin (nano-level) gold–palladium (Au–Pd) coating was applied to the samples under high vacuum for observation before imaging [83]. The average particle size and polydispersity index (PDI) were calculated by analyzing SEM micrographs using ImageJ software (Fiji Is Just ImageJ, pre-packaged version) (National Institutes of Health, Bethesda, MD, USA), measuring at least 100 individual particles to ensure statistical significance.

### 3.6. Particle Size and Polydispersity Index (PDI) Analysis

The hydrodynamic diameter and size distribution of the electrosprayed PVA/CS/Ep/VitE nanoparticles were determined using dynamic light scattering (DLS) (Zetasizer Nano ZS, Malvern Instruments, Malvern, UK). Prior to measurement, the nanoparticle samples were diluted in phosphate-buffered saline (PBS, pH 7.4) at a 1:100 (*v*/*v*) ratio and sonicated for 5 min to ensure homogeneous dispersion and an optimal scattering intensity. Measurements were performed at 25 °C with a scattering angle of 173°. The average particle size was calculated from the intensity distribution, and the polydispersity index (PDI) was recorded to assess the homogeneity of the formulation. All measurements were conducted in triplicate (*n* = 3), and the results were expressed as mean ± standard deviation.

### 3.7. Zeta Potential Measurement

The surface charge of the nanoparticles was evaluated by measuring the Zeta potential through electrophoretic light scattering (ELS) using the same 1:100 PBS dilution to maintain physiological ionic strength and prevent particle–particle interactions during electrophoretic mobility measurements. The samples were placed in a folded capillary cell (DTS1070), and the electrophoretic mobility was measured to calculate the Zeta potential based on the Henry equation. This analysis was critical to determine the colloidal stability of the system and the cationic influence of the chitosan backbone. Each sample was measured three times to ensure statistical significance.

### 3.8. In Vitro Drug Release Analysis and Kinetics Analysis

The in vitro release profiles of vitamin E (VitE) and ephedrine HCl (Ep) from the electrosprayed PVA/CS nanoparticles were evaluated using a UV-Vis spectrophotometer (752 N Plus, Türkiye). The nanoparticle formulations were immersed in 2 mL of phosphate-buffered saline (PBS, pH 7.4) and maintained at a physiological temperature of 37 °C with constant gentle agitation using a Thermo-Shaker (Yooning, Hangzhou, China). At specific time intervals (up to 48 h), the entire 2 mL of the release medium was collected for analysis and immediately replaced with an equal volume of fresh pre-warmed PBS to maintain sink conditions.

The concentration of the released bioactive agents was quantified by measuring the absorbance at their respective maximum transition wavelengths: 290 nm for vitamin E and 257 nm for ephedrine HCl. Cumulative drug release percentages were calculated based on previously established linear calibration curves for each compound.

To further elucidate the drug transport mechanism, the release data were fitted into various mathematical models, including zero-order, first-order, Higuchi, and Korsmeyer–Peppas models. The correlation coefficients (*R*^2^) were utilized to determine the best-fit model, and the release exponent (*n*) from the Korsmeyer–Peppas was analyzed to characterize the nature of the diffusion process as Fickian or non-Fickian (anomalous) transport [84].

### 3.9. Antioxidant Analysis of the Nanoparticles

The antioxidant capacity of the DL-α-tocopherol-loaded nanoparticles was evaluated using two complementary radical scavenging assays: DPPH (2,2-diphenyl-1-picrylhydrazyl) and ABTS [2,2′-azino-bis(3-ethylbenzothiazoline-6-sulfonic acid)].

#### 3.9.1. DPPH Radical Scavenging Assay

The antioxidant test was performed to verify the antioxidant property of DL-α-tocopherol in the nanoparticle. For the stock solution, 0.015 gr DPPH was dissolved in 300 mL methanol. Bioactive doped composites DL-α-tocopherol was prepared at different concentrations (1.½.¼.⅛.1/16) by diluting it with methanol. Fixed volume DPPH solution was added to the drug solutions prepared at different concentrations in the test tube (5, 10, 25, 50 µL). DPPH methanol solution was used as the control group. Ascorbic acid was prepared with DPPH at certain concentrations to create a standard reference curve. Mixtures were analyzed after 30 min of incubation. Then, 2 mL were taken from each sample and were examined at 517 nm wavelength using a UV-Vis spectrometer [80].

#### 3.9.2. ABTS Radical Scavenging Assay

As a secondary validation of the antioxidant potential, an ABTS assay was performed according to the method described by [80]. The ABTS•+ radical cation was generated by reacting 7 mM ABTS solution with 2.45 mM potassium persulfate, and the mixture was kept in the dark at room temperature for 12–16 h before use. The solution was then diluted with ethanol to reach an absorbance of 0.70 ± 0.02 at 734 nm. An amount of 100 µL of the nanoparticle dispersion was mixed with 1 mL of the ABTS•+ solution. Following 6 min of incubation, the absorbance was recorded at 734 nm using a UV-Vis spectrometer.

For both assays, the radical scavenging activity (%) was calculated using Equation (1):

(1) $$Antioxidant\;Activity(\%)=\frac{\left(Absorbance\;of\;Control\;-\;Absorbance\;of\;Sample\right)}{Absorbance\;of\;Control}\times 100$$

### 3.10. Antibacterial Analysis

The antibacterial properties of the nanoparticle formulation were investigated against *Staphylococcus aureus* (Gram-positive) and *Escherichia coli* (Gram-negative) bacteria using the disk diffusion technique. All the samples were formed into disks of almost the same size. *S. aureus* and *E. coli* bacteria were cultured at 37 °C for 24 h. Then, 0.01 mL of the above-mentioned culture medium was injected into sterilized Petri dishes. A total of 15 mL of Muller-Hinton agar (Merck) was given to each infected Petri dish. Disks were placed on the solid agar medium by gently pressing. The treated Petri dishes were incubated at 37 ± 1 °C for 24 h. The inhibitory zones developed on the medium were finally measured. Antibacterial activity experiments were performed in triplicate for each test strain and average measurements were calculated. Four different antibacterial tests were performed for the four different components of the formulation PVA/CS, PVA/CS/Ep, PVA/CS/VitE, PVA/CS/Ep/VitE [83].

### 3.11. Statistical Analysis

All statistical data analyses were performed via ANOVA using GraphPad Prism version 10 software (GraphPad Software Inc., San Diego, CA, USA). The values are as follows: mean ± standard deviation (SD) and statistical differences were analyzed by one-way ANOVA and Tukey and Dunnet multiple comparison tests. A *p* value of <0.05 was considered statistically significant in all cases.

## 4. Conclusions

In this study, an innovative nanoparticle design compatible with intranasal application for the treatment of narcolepsy, which currently has limited diagnostic and therapeutic options, is presented. The design in question consists of a **PVA/CS** mixture polymer structure and an active ingredient combination of DL-α-tocopherol and ephedrine. FDA-approved active ingredients and polymers were incorporated to create a design that will provide delivery to brain regions such as the hypothalamus, which are related to the disease and sleep mechanism, via the nose-to-brain axis using an advanced fabrication technique called electrospraying. Comparative characterization studies performed show that a large number of nanoparticles were successfully formed from the designed formulation.

Morphological findings confirm that particles with controlled release potential and high bioavailability were synthesized. The drug formulation showed a stable in vitro release profile and proved its antibacterial properties against Gram-positive and Gram-negative bacteria. FT-IR results reassured a stable and bioavailable chemical structure. Finally, the antioxidant test results indicate that the formulation produced has the potential to inhibit the formation of reactive oxygen species, which is the natural outcome of the molecular pathophysiology of narcolepsy and can stop not only the symptoms but also the disease progression.

This study highlights the significant potential of intranasally compatible nanoparticle formulations in diseases of the central nervous system in terms of regional targeting and controlled release with minimal systemic side effects through noninvasive application. In addition, the bioactive components used, ephedrine and DL-α-tocopherol, are promising in terms of the treatment of narcolepsy.

## Figures and Tables

**Table 1:** In vitro release kinetic parameters of vitamin E and Ephedrine from PVA/CS nanoparticles.

Drug	Zero-Order (*R*^2^)	First-Order (*R*^2^)	Higuchi (*R*^2^)	Korsmeyer–Peppas (*R*^2^)	*n* Value	Release Mechanism
Vitamin E	0.584	0.626	0.957	0.971	0.476	Anomalous Transport
Ephedrine	0.421	0.714	0.886	0.987	0.575	Anomalous Transport

**Table 2:** Detailed formulation compositions and solvent ratios for the electrosprayed nanoparticles.

Formulation Code	PVA (mg)	CS (mg)	Ephedrine HCl (mg)	Vitamin E (mg)	Solvent System (Water: EtOH:AcOH)
PVA/CS	2250	250	-	-	82:16:2 (*v*/*v*/*v*)
PVA/CS/Ep	2250	250	25.0	-	82:16:2 (*v*/*v*/*v*)
PVA/CS/VitE	2250	250	-	25.0	82:16:2 (*v*/*v*/*v*)
PVA/CS/Ep/VitE	2250	250	7.5	17.5	82:16:2 (*v*/*v*/*v*)

## Data Availability

The study is completely new, and all data and photos are shared in this study.
